# The Visible Behaviour of Drowning Persons: A Pilot Observational Study Using Analytic Software and a Nominal Group Technique

**DOI:** 10.3390/ijerph17186930

**Published:** 2020-09-22

**Authors:** Aida Carballo-Fazanes, Joost J.L.M. Bierens, the International Expert Group to Study Drowning Behaviour

**Affiliations:** 1 CLINURSID Research Group & Santiago de Compostela’s Health Research Institute (IDIS), Universidade de Santiago de Compostela, 15705 Santiago de Compostela, Spain; 2 Research Group Emergency and Disaster Medicine, Vrije Universiteit Brussel, 1090 Brussels, Belgium; jbierens@euronet.nl

**Keywords:** observation, video analysis, bystander, nominal group technique, LINCE software, water safety

## Abstract

Although drowning is a common phenomenon, the behaviour of drowning persons is poorly understood. The purpose of this study is to provide a quantitative and qualitative analysis of this behaviour. This was an observational study of drowning videos observed by 20 international experts in the field of water safety. For quantitative analysis, each video was analysed with Lince observation software by four participants. A Nominal Group Technique generated input for the qualitative analysis and the two principal investigators conducted a post-hoc analysis. A total of 87.5% of the 23 videos showed drowning in swimming pools, 50% of the drowned persons were male, and 58.3% were children or teenagers. Nineteen persons were rescued before unconsciousness and showed just the beginning of downing behaviour. Another five were rescued after unconsciousness, which allowed the observation of their drowning behaviour from the beginning to the end. Significant differences were found comparing both groups regarding the length of disappearances underwater, number, and length of resurfacing (resp. *p* = 0.003, 0.016, 0.005) and the interval from the beginning of the incident to the rescue (*p* = 0.004). All persons drowned within 2 min. The qualitative analysis showed previously suggested behaviour patterns (immediate disappearance *n* = 5, distress *n* = 6, instinctive drowning response *n* = 6, climbing ladder motion *n* = 3) but also a striking new pattern (backward water milling *n* = 19). This study confirms previous assumptions of drowning behaviour and provides novel evidence-based information about the large variety of visible behaviours of drowning persons. New behaviours, which mainly include high-frequency resurfacing during a struggle for less than 2 min and backward water milling, have been recognised in this study.

## 1. Introduction

Visualization of a drowning person is a low-frequency event and there are scant published data on the behaviours of drowning persons. What actually occurs most of the time is that the initial moment when drowning starts is missed [1,2,3,4]. The relatively scarce number of real-life observations is certainly one of the main reasons why there is limited evidence-based understanding of the behaviour of a person in the process of drowning. Although each year at least 372.000 persons die from drowning worldwide, it is a neglected issue. There is a great need for research, covering all aspects of drowning reduction. This includes understanding how to notice a person who is drowning [5].

For many years it has been assumed that a person in the water will scream and wave for help when drowning. This is shaped and reinforced by popular lay-media depictions of drowning [6,7]. Pia [8,9] studied real-situation film footage to form the basis for the “Instinctive Drowning Response” (IDR) theory (eyes fixed to shore, horizontal arm movements just underwater). The IDR is currently the most accepted concept for visible drowning behaviour [10,11,12,13,14].

Theoretical concepts have also been explored to better classify drowning behaviour, namely the 4 W-model, C-zones framework and SENTINEL [1,15,16,17,18]. The 4 W-model emerges from an analysis of videos with drowning incidents videos and interviews with persons who had experienced a non-fatal drowning. The analysis concludes that rescuer characteristics, causality characteristics, location, and general circumstances determinate the outcome of drowning [1]. The C-Zones framework explains how a drowning tends to progress through seven zones (stages): comfort, concern, crisis, critical, cardiopulmonary resuscitation, coma, conclusion [15]. The SENTINEL model is a system for drowning risk stratification based on the analysis of drowning behaviour video footage [16].

The large increase in footage provided via security cameras, closed-circuit television cameras, social media, and lawsuits, introduces the possibility of confirming or adapting the existing theories surrounding drowning behaviour and to determine if there is a wider variety of drowning behaviours than currently assumed.

In this study, publicly available video footage has been reviewed with the aim of systematically registering and analysing visible drowning behaviours. A more comprehensive and complete understanding of drowning behaviour will have an important impact on the ability to recognize and respond to a drowning event.

## 2. Materials and Methods

At the 2019 World Conference on Drowning Prevention (WCDP), in Durban, South Africa, a group of persons with diverse expertise in drowning prevention, rescue, and treatment was invited to participate in a drowning behaviour study by means of an invitation-only meeting.

Persons with practical expertise in the field of water safety were selected from the members of the International Drowning Researchers’ Alliance (IDRA) by the primary authors (A.C.-F., J.J.L.M.B.). In addition, the members of the Medical Committee and Rescue Commission of the International Life Saving Federation (ILS), as well as experts who had published or presented papers on this issue at conferences were contacted. Invitees were also asked to provide additional leads, who were contacted. Initially, over 80 experts showed interest in participating, although only 20 were able to attend the WCDP2019 and to participate in video viewing sessions, data collection, and analysis during the first viewing session; 16 were able to participate during the second viewing session; and 13 persons were able to attend the two adapted Nominal Group Technique sessions. Of the 20 participants from eight different countries (Australia, Brazil, United Kingdom, Greece, New Zealand, Portugal, Spain and USA), 16 were men (80%). The mean age (SD) was 43.6 (11.2) years and the years they worked in the drowning field averaged 22.7 (12.2) years. Attendance decreased at each session because of logistic reasons and fatigue due to recent intercontinental travelling. In total, the meeting lasted 11 h (including video sessions, preparing and solving the technical aspects and regular breaks).

**Figure 1:**
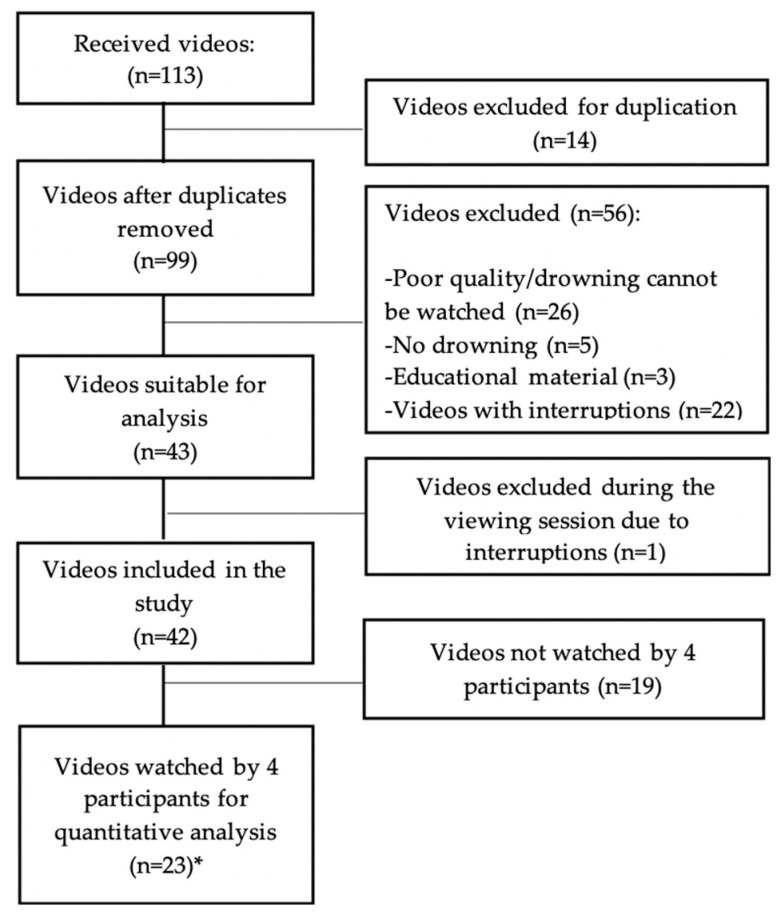
Video selection process for the quantitative analysis. * Each video was watched only one time by each of the four participants.

At the same time that the experts had been invited to consider participation, the same experts were requested to submit publicly available videos that could be considered to be used for the study. One hundred and thirteen videos were submitted by 13 persons. Finally, 43 were deemed eligible by A.C-.F. and C.A-.G. for the analysis of the drowning behaviour as they showed the beginning and end of the drowning process without interruptions and were of sufficient quality to be analysed (Figure 1).

In health science, drowning is defined as the process of respiratory impairment as a result of submersion or immersion in a liquid medium [19]. In a more common sense, the term drowning is used to describe persons who are in distress, struggling, or at risk of drowning, even before there is respiratory impairment. For the purpose of this study, drowning videos were used in the context of the common usage of the term drowning.

In the first viewing session, the 20 participants were allocated at random into 10 teams of two participants. The 43 videos were at random grouped in eight sets of five videos and one set of three videos. Nine teams of two participants were randomly allotted to one set of videos, the tenth group was appointed as jury in case the members of any of the other groups did not agree. During the second viewing, 16 participants were allocated at random into eight different teams of two participants, taking care that no group would be identical to the first session and no participant would view the same video a second time; the supervising team (A.C.-F., J. J.L.M.B.) had the function of jury. 

To avoid bias and peer pressure, each participant independently watched one video at a time and noted the observations on a standard registration form, adapted and expanded after being used in another study on the topic [20]. The form included information about age, gender, location, time moments, activity in the water, water movements, use of flotation devices, the presence of others in and out the water who detected and rescued the drowning person, position changes in the water, drowning concepts (defined as Instinctive Drowning Response, waving, none of them or unknown) and origin of footage. The videos were assessed without audio to avoid distracting the other observers in the same room.

The data on these forms were compared between the two participants in the same team who watched the same video. If needed, differences were discussed until consensus was reached, with the help of a jury if required. Next, common observations were first recorded on a consensus registration form and imported in the LINCE software program (LINCE observation software; v.1.2.1) [20] by both participants of the team together. 

The first viewing session took 90 min, the second 60 min. Most teams were unable to view all videos allotted to them. At the end of the viewing periods, 23 videos had been watched by two teams (meaning that each of these videos was watched by four participants), eight videos had been watched by one team (watched by two participants) and 11 videos had not been viewed. One video had gaps and repetitions in the footage and was excluded during the viewing process.

Two researchers (A.C.-F., J. J.L.M.B.) controlled all the paper forms and checked the quantitative data included in the software of the 23 remaining videos for accuracy. Despite uniformity being reached between the observations within each team, when the data collected from the teams about the same drowning were compared, it was clear that there were differences in the observations between two teams regarding the same video. These differences were partly due to minor issues (e.g., age category young adult vs. old adult), and unrealistic observations (e.g., to register each period of submersion when a person resurfaced several times for few seconds during a life-threatening struggle). All differences were solved by A.C.-F. and J. J.L.M.B. by combining categories (all adults in one group), or by redefining specific parameters such as the total number of re-surfacing within the full period of the struggle. When time differences were less than 10 s, the average was taken. When more than 10 s of the time difference, A.C.-F. and J. J.L.M.B. watched the video again and decided by consensus. 

After the two viewing sessions for quantitative analysis, there were another two viewing sessions for qualitative analysis using an adapted Nominal Group Technique (NGT); six participants jointly and uninterrupted watched a series of 20 videos of 22 children who drowned in pools (length: 25:35 min), and another group of seven participants watched a series of 11 videos with 16 adults who drowned in pools (*n* = 3), lake, river or canal (*n* = 5) and sea (*n* = 3) (length: 27:17 min). In a second NGT session, each group watched the alternative series of videos. Eleven videos were not included in the NGT sessions. These were typical uniform drowning incidents of toddlers in floating rings who simply turned upside down; situations where the rescuer also drowned during contact with the drowning victim; or because of a lack of time. While watching the video series, the participants were invited to note and register in silence the drowning behaviour as a basis for grounded theory development. Then, the participants discussed their observations within the group, focusing most on the recognition of any known or newly recognised visible drowning behaviour pattern. The observations and the comments made during the sessions were collected. Based on the information from the individual experts during the nominal group technique, an aggregated understanding of the various aspects of the drowning behaviour was acquired. Using this information, the principal investigators (A.C.-F. and J. J.L.M.B.) analysed in a separate session, 2 months after the sessions in Durban, all 42 videos with 52 drowned persons and were able to study the several details and patterns mentioned during the NGT session. 

### 2.1. Software Tools and Statistical Analysis

The quantitative data of the viewing sessions were entered into a free LINCE software package. LINCE software, only available to Windows operating system, is commonly used to analyse behaviour in sports [21,22,23] and allows simultaneous viewing and recording of behaviours while watching videos on a computer screen. The software also allows data to be exported in different formats for analysis. The software had been tested in a previous drowning study [20]. The participants had been introduced to the software before arriving at the meeting and briefed at the beginning of the meeting. Instructions and support were available at all times during the meeting. 

Data were analysed with the statistical software IBM SPSS Statistics for Mac (v. 25.0, Armonk, NY, USA: IBM Corp., 2017). A descriptive analysis of the variables was carried out to identify the most important parameters of the drowning persons and the drowning process. The categorical variables were expressed as absolute frequencies and relative frequencies, and the scale variables were expressed as median and interquartile range. The normality of the data was checked through the Shapiro–Wilk test. Mann–Whitney test was applied to compare continuous data between persons rescued in time vs. the persons who appeared on the video to be unconscious by the end of the drowning process. To compare the different rescue options and the timing of the rescue, the non-parametric test of Kruskal–Wallis was used. A linear regression model was used to assess the relationship between the number and duration of resurfacings. An alpha level of 0.05 was used for all analysis.

### 2.2. Ethical Committee

The ethical objections and the benefit of the results of the study, using publicly available video footage, have been carefully considered. Ethical approval by the Faculty of Education and Sport Sciences (University of Vigo, Pontevedra, Spain) for the study was received (Code 03–1019). All participants read an information sheet and signed an informed consent form, a conflict of interest form, and a declaration of confidentiality. Participants were also informed that watching the videos might pose emotional or psychological consequences.

## 3. Results

### 3.1. Quantitative Analysis

In the 23 videos analysed, there was a potential sample of 25 drowning persons. However, upon closer examination, one drowning person in one of the videos with two victims was excluded since this person was pushed underwater while attempting to rescue the person who was in the water first. Therefore, 24 drowned persons from 23 videos were included in the analysis. A description of persons and circumstances is shown in Table 1. The majority of videos depicted children or teenagers (58.3%). The most frequent location of drownings in the study was outdoor swimming pools (58.3%).

Table 2 shows time-related variables expressed as median and interquartile range into two groups: persons rescued in time (*n* = 19) and persons who appeared on the video to be unconscious by the end of the drowning process (*n* = 5).

**Figure 2:**
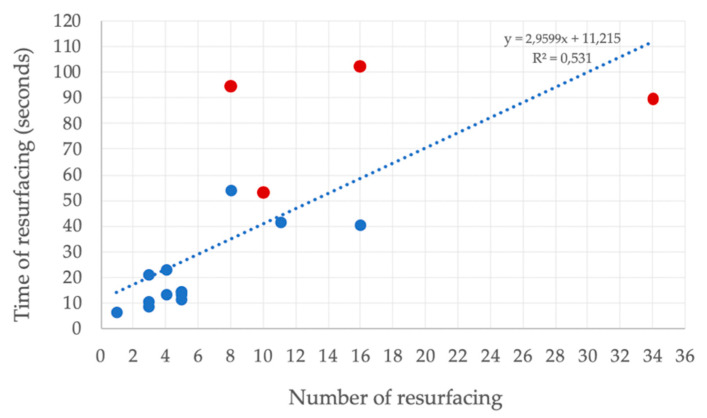
Number of resurfacings over the period of time of the survival struggle (seconds) for all drowning persons. Blue dots represent persons rescued in time and red dots represent persons appeared on the video to be unconscious by the end of the drowning process (*p* < 0.001).

Figure 2 shows the linear regression model between the duration of the struggle and the number of resurfacing events (R^2^ = 0.531; F = 16.957, *p* = 0.001).

Although there was no significant difference in rescue times found when the rescue was performed by persons outside the water, persons in the water, or lifeguards (63 (9.5–137); 48 (18–214); 17 (12–26) seconds of median (interquartile range) respectively; *p* = 0.513), the lifeguards rescued all persons within 30 s.

### 3.2. Qualitative Analysis

**Figure 3:**
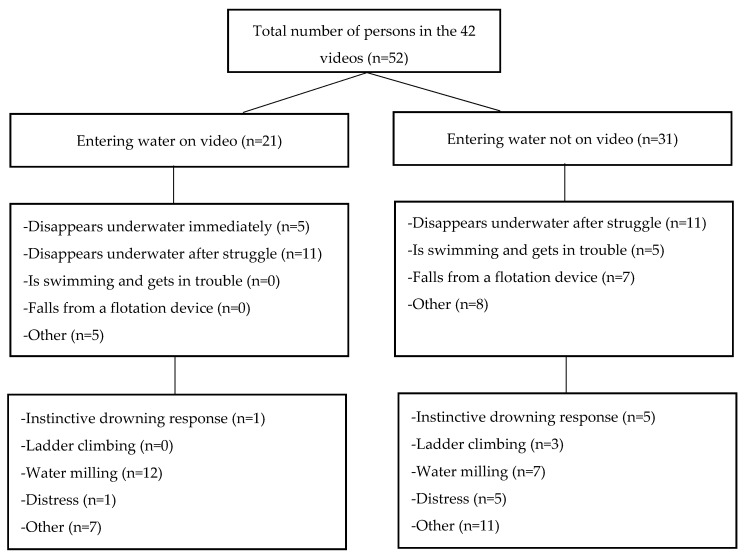
Drowning behaviour of 52 persons in 42 videos based on the observations as an input of the Nominal Group Technique sessions.

During the two adapted NGT sessions, it was observed that the drowning process usually started either immediately when entering the water, after some struggle or after a period of swimming. The visual drowning behaviour showed a broad range of behaviours; most of them could be clearly described (Figure 3). The pattern of drowning behaviour in children, who all drowned in pools, was much more homogenous than the pattern in adults, who drowned in various settings. A behaviour that matched the IDR was recognised by the observers as well as a modified version with “climbing ladder motion”. A typical behaviour recognized by observers was similar to one of the components of the IDR—the non-voluntary control movement consisting of extending the arms laterally and beginning to press down on the surface of the water to try to breathe—but was more forceful and with clear splashes of water while the arms were rotated fiercely backwards in an attempt to keep the front of the head out of the water. There was no evidence in any of the videos of persons waving for help and there were no indications that any of the drowned persons tried to shout or scream.

Other remarkable behaviours were identified and are reported for their potential relevance to future research. These include (of the total number of drowning persons, *n* = 52):

▪ Several children who drowned seemed to be playing alone (*n* = 19).
▪ Several children seemed to jump into the water with great confidence to discover that they could not manage the situation in the water (*n* = 9).
▪ People seemed to be looking at the person who is drowning without taking action (*n* = 13). In some of these videos, the distance between them was less than an arm-reach.
▪ One person in trouble with another person, or with a group, causes them all to drown (*n* = 5).
▪ Toddlers in floating rings who simply turned upside down (*n* = 3).
▪ One child initially seemed to be playing in the water by tumbling head forward. After some time, the movements remain identical but less intentional. At the end, the child does not move at all and remains motionless floating for around 3 min just under the surface before being taken out by a pool visitor.
▪ One adult, while swimming, suddenly disappears underwater while his swimming movements become weaker. Weak and uncoordinated underwater movements continue for almost 4 min before all movements stop. In another swimmer, the same happened initially but this person was rescued. Both situations occurred in a full-sized swimming pool and it is highly unlikely that the movements were caused by underwater currents.

## 4. Discussion

In this observational study, the drowning of 24 persons captured on video was analysed by five panels of four experts to extract quantitative data. In addition, grounded on the observations during two Nominal Group Technique sessions, a qualitative analysis of the visible drowning patterns in 52 persons was conducted. To the best of our knowledge, this is the first time that drowning has been investigated in a way that allows a reproducible and evidence-based evaluation and understanding of visible drowning behaviour.

The video captured five persons who at the end of the video appeared to be unconscious. Their attempts not to drown had a mean duration of 92 s, during which they resurfaced 13 times (Table 2). The drowning behaviour had not been observed or recognized although, in each video, several persons were inside and outside the water. Our observations suggest that there is, at most, 2 min to identify and recognise a person who drowns. Figure 2 shows that there is an association between the time of the struggle and the number of resurfacings for persons who attempt not to drown; on average resurfacing occurs with a high frequency of six times in 30 s. The other five drowned persons, where the entering of the water was captured on video, disappeared immediately underwater and showed no visible behaviour at all.

No person was observed to wave or shout for help. Other studies have also suggested that waving and shouting for help does not occur during life-threatening drowning events. The loss of buoyancy during exhalation of air while screaming, and stopping supportive arm movements while raising one or both arms, both impede swimming and floating [1,6,17,18,24].

The most common and only description of the drowning behaviour is the IDR concept which was first described in 1974 and has since then been included in all major lifeguard manuals. The description is based on the observations by one lifeguard, Frank Pia, of 13 persons who drowned at Orchard Beach (New York) during recreational activities. The persons entered the ocean in front of the lifeguard in water that was beyond their depth [8,9]. The response includes a group of negative characteristics: the person is unable to call out for help, cannot wave for help, cannot voluntarily control their arm movements, and is unable to move in a horizontal or diagonal direction. The struggle at the surface of the water lasts between 20 and 60 s. A positive characteristic is non-voluntary controlled movements that consist of extending the arms laterally and of pressing down on the surface of the water to try to breathe [8,9,25]. Sometimes “climbing ladder motion” is added to this, although no evidence for this could be found [26]. Pia also mentions the behaviour of people in distress: voluntary control of movements that consist of moving the arms and legs to keep the mouth above the surface of the water [25].

In our videos, using observers with knowledge and experiences in this field, the IDR behaviour was observed nine times, including three persons with a ladder motion. Distress was observed in another six persons. A new phenomenon that appears as typical behaviour was recognized in 19 persons: the arms were extended laterally and forcefully rotating backwards in an attempt to keep the front of the head out of the water. Clear splashes of water were visible. This appeared to be what we would refer to as backwards water milling.

In five videos, people drowned in an attempt to rescue another person. A badly performed rescue implies a risk that can result in the “AVIR syndrome” (aquatic victim instead of rescuer) [14,27,28,29]. This observation again emphasises the importance of community-wide teaching of rescue skills [30].

This analysis revealed additional remarkable observations in 18 persons. Some of the remarkable observations were made once (i.e., a child and two adults that showed ineffective movements underwater for several minutes), while other observations were made several times. Additionally, observations were made about other persons in and outside the water. In 13 persons showing clearly visible drowning behaviour or visibly floating motionless, bystanders seemed to ignore the problem happening within arm’s reach [26]. Lifeguards also failed to recognize the signs of struggle, sometimes because they were distracted or were looking in another direction while the drowning was happening. Others may have been untrained, and unable to recognise the behaviour [2,9,31,32,33,34,35,36,37,38,39,40,41]. This study was motivated by the increasingly recognised need to more deeply understand and describe the visible behaviour of drowning persons [42]. There is growing evidence that early recognition of the drowning event and early rescue are the most important factors that determine the prognosis [43,44,45].

This study was able to describe several aspects of the visible behaviour of drowning persons by focusing strictly on the movements of the victims regardless of the cause of drowning. In addition to the IDR, this study identified new behavioural patterns—repeated, and sometimes prolonged, resurfacing for a maximum of two minutes as well as backwards water milling—as typical visible drowning behaviours that have not been described before.

All these observations deepen our understanding of the visible drowning behaviour, revealing most of all that it is more complex and varied than currently expected. In 17 of 42 persons included in the qualitative analysis, the behaviour did not fit into the traditional or newly recognised behaviours. In some videos it was “mysterious” what actually happened [36]. It has been suggested that some of this behaviour may be related to a physiological response to a stressful situation [46].

Prevention of fatal and non-fatal drowning will most likely ameliorate when both the general population, who may need to intervene as a bystander, and those with a formal rescue task are educated about the various visible drowning behaviours. However, before the population or lifeguards are educated on the larger variety of visible drowning behaviours, more research is needed, based on more video analysis with a larger number of complete drowning behaviours and a more detailed description of the variety of the visible behaviour. This exercise should continue, as a minimum, until no new drowning behaviours are observed.

## 5. Limitations

The videos analysed in the study were based on a subset of videos selected on convenience, because they were available as YouTube files, and on the quality and feasibility of the video footage, that allowed the analysis needed for this study. Videos recorded by CCTV cameras were overrepresented (71%), and therefore the majority of the recorded drownings occurred in swimming pools. In some countries, home and backyard pools drownings occur frequently [47,48]. However, in most countries, pool drownings are responsible for a minority of fatal and non-fatal drownings [31,38,48,49].

The limited number of videos did, for example, not allow for categorizing the drowning behaviour by age groups. Some experts at the meeting believe that this behaviour varies among the different age groups. A more comprehensive subset analysis of these and other variables is required in future studies.

The participants were selected because of their expertise. Only those experts attending the WCDP2019 could participate and an experienced and a diverse group was present at the meeting. It may have been that other selected experts may have made different observations. Laypersons may also have had different interpretations of the behaviours. This may have resulted in a larger variety of observations, but it is unlikely that the current findings would be rejected.

The study design was not thoroughly tested before implementation, which led to unforeseen issues during the study phase. The setting of the meeting and the lack of any additional information on the drowning setting may also have affected the quality of the observations. Not all experts could attend the full eleven hours of the meeting and they were not able to observe all videos. This, added to the novelty of this methodology for analysing the drowning behaviour, led to the need for diligent methodological improvisation, as described in the methods.

For a better understanding, it is important that future studies include a larger variety of videos, with a greater diversity of location and ages, and include a wider range of experts and laypersons in a more accommodating setting to perform the analysis.

Although the LINCE system has been used for the analysis of movements of several sporting activities, the system showed some limitations when used to analyse the behaviour of drowning persons. The spontaneous actions or movements that occur during drowning are more subjective to identify than the more intentional movements in sports. This makes it more difficult to quantify the drowning movements. Some videos were recorded with low frame rates in relation to the high speed of the drowning behaviour or with poor quality, making it difficult to analyze some parameters. Nevertheless, the software allowed systematic, objective, and reproducible measures of the movement that occurred. The software LINCE, although simple, was unknown to most of the participants and a steep learning curve was observed after the meeting had started.

Even given these limitations, the authors believe that this pilot study provides critical, original and useful information and that these data are the best, to date, for describing the drowning behaviour that should be publicized. It also describes a new methodology for analysing and understanding the existence of a much wider range of drowning behaviour than was previously thought to exist. Future research directions may also be highlighted.

## 6. Conclusions

Poor understanding of the visible drowning behaviour is one of the most relevant knowledge gaps in the field of drowning prevention research. This study provides new information, collected in a systematic and reproducible way with a maximum avoidance of bias.

The visible behaviour of drowning persons is much more complex than previously described, can present with many variations, and there is ample time to recognize that a person is drowning. The full pattern of visible behaviour of drowning victims is most likely still largely unknown and requires further investigation. The current findings will assist members of the public, lifeguards and other water safety professionals, as well as researchers and industries involved in automatic drowning detection systems [50,51,52,53,54,55,56,57], to identify a drowning person and understand the importance of looking for these behaviours amongst water users [58].

## Figures and Tables

**Table 1:** Description of the drowning persons and circumstances.

		*n* (%)	
Age	Baby	4	(16.7)
	Child/Teenager	14	(58.3)
	Adult	6	(25)
Gender	Male	12	(50)
	Female	5	(20.8)
	Unknown	7	(29.2)
Location	Home swimming pool outdoor	2	(8.4)
	Swimming pool indoor	5	(20.8)
	Swimming pool outdoor	14	(58.3)
	Open water	3	(12.5)
Water movements	Water is moving	2	(8.3)
	Water stands still	22	(91.6)
Floatation device	Without flotation device	17	(70.8)
	With flotation device	4	(16.7)
	Other	3	(12.5)
Presence of lifeguard	Yes	8	(33.3)
	No	16	(66.7)
Presence of others in the water	Nobody	8	(33.3)
	Less than 5 people	6	(25)
	Between 5 and 25 people	6	(25)
	More than 25 people	4	(16.7)
Presence of others outside the water	Nobody	1	(4.2)
	Less than 5 people	14	(58.3)
	Between 5 and 25 people	8	(33.3)
	Unknown	1	(4.2)
Drowning detected by	Person in the water	7	(29.2)
	Person outside the water	11	(45.8)
	Lifeguard	6	(25)
Rescue	Self-rescue	1	(4.2)
	Person in the water	7	(29.2)
	Person outside the water	9	(37.5)
	Lifeguard	7	(29.2)
Footage	CCTV camera	17	(70.8)
	Accidentally captured by camera	2	(8.3)
	Intentional captured by camera	5	(20.8)

**Table 2:** Description of variables in rescued persons (*n* = 19) and not rescued before unconscious (*n* = 5).

	Persons Rescued			Persons Not Rescued; Appeared on the Video to Be Unconscious by the End of the Drowning Process			*p*-Value **
	Median (Q1–Q3)	Range	*n*	Median (Q1–Q3)	Range	*n*	
Number of disappearances	5 (2–6.5)	1–16	17	10 (4.5–25)	1–34	5	*p* = 0.080
Length of disappearances (seconds)	13 (7–20.5)	0–54	17	90 (43.5–98)	34–102	5	*p* = 0.003
Number of resurfacing	5 (3–6.5)	1–16	13	13 (8.5–29.5)	8–34	4	*p* = 0.016
Length of resurfacing (seconds)	14 (10.5–31.5)	7–54	13	92 (62.3–100)	53–102	4	*p* = 0.005
Time of movements of the person above the water stop due to rescue (seconds)	17 (14–58)	7–160	15				
Time of movements of the person above the water stop spontaneously (seconds)				106 (96.3–125.5)	106–132	4	
Time of final disappearance underwater (seconds)				106 *	34–132	3	
Duration of the visible drowning behaviour above the water until the rescue (seconds)	14.5 (10.3–44.5)	4–81	16				
Duration of the visible drowning behaviour above the water until this spontaneously stopped (seconds)				94.5 (80.5–95.8)	76–96	4	
The interval from the beginning of the incident to the final disappearance (seconds)				94 *	6–95	3	
The interval from the beginning of the incident to the rescue (seconds)	15 (10–45)	4–154	19	229 (89.8–261.8)	52–264	4	*p* = 0.004

Q1: quartile 1; Q3: quartile 3. *n*: number of drowning persons in which the variable occurs. * Unable to calculate interquartile range *n* = 3. ** Mann-Whitney test.

## Appendix A

The following experts are members of the International Expert Group to Study Drowning Behaviour:

▪ **Cristian Abelairas-Gómez (CA-G**), Faculty of Education, University of Santiago de Compostela, Santiago de Compostela, Spain;
▪ Stathis Avramidis, Greek Lifesaving Sports Association, Athens, Greece;
▪ **Shayne Baker**, Royal Life Saving Society Australia, Sydney, Australia;
▪ **Roberto Barcala-Furelos**, Faculty of Education and Sport Sciences, REMOSS University Research Group in Lifesaving, University of Vigo, Pontevedra, Spain;
▪ **Rachel Griffiths**, Aquatic Safety Research Group, State College, Pennsylvania, United States;
▪ **Tom Griffiths**, Aquatic Safety Research Group, State College, Pennsylvania, United States;
▪ **Gareth Hedges**, H2O Safety Consulting, Durham North Carolina, USA;
▪ **William Koon**, School of Biological, Earth and Environmental Sciences, University New South Wales Sydney, Sydney, Australia;
▪ **Dan Kwaczynski**, Australian Leisure Facilities Association, *Brisbane, Queensland, Australia;*
▪ **Ross Macleod**, Royal National Lifeboat Institution, Dorset, Great Britain;
▪ **Adrian Mayhew**, Surf Life Saving, Exeter, Great Britain;
▪ **Patrick Morgan**, Extreme Environments Laboratory, School of Sport, Health & Exercise Science, University of Portsmouth, Portsmouth, Great Britain;
▪ **Nick Mulcahy**, Surf Life Saving New Zealand, Lower Hutt City, Wellington, New Zealand;
▪ **Liliana Oliveira**, Safer in Water, Guimarães, Portugal;
▪ **Luis-Miguel Pascual-Gómez**, Segovia Life Saving School, Segovia, Spain;
▪ **Ana Catarina Queiroga**, Institute of Public Health of the University of Porto, Porto, Portugal;
▪ **Craig Roberts**, Royal Life Saving Society Australia, Sydney, Australia;
▪ **Justin Sempsrott**, Lifeguards Without Borders, Kuna, Idaho, USA;
▪ **Jennifer Smith**, Institute of Sport, University of Chichester, Chichester, Great Britain;
▪ **Leonardo Springer**, Higher Institute of Education and Sciences (ISEC) Lisboa, Portugal;
▪ **David Szpilman**, Brazilian Life Saving Society (SOBRASA), Rio de Janeiro, Brazil;
▪ **Michael J. Tipton**, Extreme Environments Laboratory, School of Sport, Health & Exercise Science, University of Portsmouth, Portsmouth, Great Britain;
▪ **Evangelos Tsampazis**, Lifeguard Academy of Northern Greece, Thessaloniki, Greece.
